# Therapeutic Alliance, Treatment Intensity, and Symptom Change in Post-traumatic Stress Disorder: A Retrospective Study of Eye Movement Desensitization and Reprocessing-Centered Psychotherapy

**DOI:** 10.7759/cureus.103603

**Published:** 2026-02-14

**Authors:** Ron Gabriel A Peji, Jeannie A Perez

**Affiliations:** 1 College of Professional and Graduate Studies, De La Salle University, Dasmariñas, PHL; 2 Center for Psychotraumatology, Livence Mental Health, Silang, PHL

**Keywords:** eye movement desensitization and reprocessing, posttraumatic stress disorder, symptom change, therapeutic alliance, trauma-focused psychotherapy, treatment intensity

## Abstract

Background

Eye movement desensitization and reprocessing (EMDR)-centered psychotherapy is an evidence-based treatment for post-traumatic stress disorder (PTSD), yet symptom trajectories during routine outpatient care vary considerably. Process factors such as therapeutic alliance and treatment intensity may influence outcomes, but their independent and combined contributions in real-world clinical settings remain unclear. This study examined the relationships among therapeutic alliance, treatment intensity, and PTSD symptom change in a naturalistic outpatient context.

Methods

This single-center retrospective cohort study analyzed routinely collected clinical data from adults with a clinician-confirmed chart diagnosis of PTSD based on the Diagnostic and Statistical Manual of Mental Disorders, Fifth Edition (DSM-5) criteria who completed EMDR-centered psychotherapy at a single outpatient psychological clinic in the Philippines between 2022 and 2025. PTSD symptoms were assessed using the PTSD Checklist for DSM-5 (PCL-5) at baseline, mid-therapy, and post-treatment. Therapeutic alliance was measured at mid-therapy using the five-item Agnew Relationship Measure-5 (ARM-5). Treatment intensity was defined as the total number of completed psychotherapy sessions. Linear regression and moderation analyses examined associations between alliance, treatment intensity, and symptom change, adjusting for age, sex, trauma type, and therapy type.

Results

The sample included 138 patients. Mean PTSD symptom scores decreased from 43.7 at baseline to 37.8 at mid-therapy and 30.0 at post-treatment. Therapeutic alliance did not predict early symptom change (baseline to mid-therapy; β = −0.01, p = 0.878) but significantly predicted overall symptom reduction (baseline to post-treatment; β = 0.61, p < 0.001; R² = 0.245) and late-phase improvement (mid-therapy to post-treatment; β = 0.62, p < 0.001; R² = 0.207). Treatment intensity significantly predicted symptom reduction from baseline to post-treatment (β = −0.21, p < 0.001; adjusted R² = 0.251) and from mid-therapy to post-treatment (β = −0.26, p < 0.001). Moderation analyses showed no significant interaction between therapeutic alliance and treatment intensity across treatment phases (all p > 0.10), indicating independent effects.

Conclusions

In routine outpatient EMDR-centered psychotherapy, therapeutic alliance and treatment intensity independently predict PTSD symptom improvement. Alliance appears most consequential after initial treatment engagement, while greater session completion supports sustained symptom reduction. These findings underscore the importance of fostering a strong working alliance beyond early sessions and promoting adequate treatment exposure to optimize outcomes in real-world trauma-focused care.

## Introduction

Post-traumatic stress disorder (PTSD) is a prevalent and impairing mental health condition that is now routinely treated using trauma-focused psychotherapies [1]. Contemporary clinical guidelines consistently position eye movement desensitization and reprocessing (EMDR) as a first-line intervention for adults with PTSD, alongside trauma-focused cognitive behavioral therapies (TF-CBTs) [1,2]. Comparative meta-analyses further demonstrate that EMDR and TF-CBT yield the most reliable symptom reductions and remission gains at post-treatment, with effects maintained at short-term follow-up [3,4]. While these findings establish EMDR as an efficacious intervention, they also raise important questions regarding how symptom change unfolds during treatment in routine clinical settings, beyond average group effects observed in controlled trials.

Accumulating evidence indicates that PTSD symptom trajectories during psychotherapy are heterogeneous rather than uniform [5,6]. Change-based research has consistently identified subgroups of patients who show rapid improvement, gradual response, or minimal change over the course of evidence-based treatments, with important implications for clinical decision-making and treatment planning [5,6]. For example, latent class analyses among veterans receiving cognitive processing therapy (CPT) have demonstrated distinct symptom-change patterns, with baseline severity and comorbidity predicting class membership [5]. Similar heterogeneity has been observed in intensive CPT programs, where patients exhibited varied rates and magnitudes of improvement despite standardized treatment delivery [6]. Understanding these patterns within EMDR-centered psychotherapy may help identify which patients benefit early, which require extended treatment, and which may need adaptive interventions.

Among psychotherapy process variables, therapeutic alliance, i.e., the collaborative working relationship between client and therapist focused on shared goals, tasks, and emotional bond, has consistently emerged as one of the most robust and transdiagnostic predictors of treatment outcomes [7-9]. Large-scale meta-analyses across psychotherapy modalities demonstrate a moderate and reliable association between alliance and symptom change [7,8]. Importantly, a PTSD-specific meta-analysis confirmed that therapeutic alliance significantly predicts reductions in PTSD symptoms across treatment modalities and delivery formats, with effect sizes comparable to or exceeding those observed in general psychotherapy samples [9]. These findings suggest that alliance is not merely a nonspecific correlate of improvement but a clinically meaningful process variable that may shape treatment response, particularly during the active phases of trauma-focused therapy.

Treatment intensity, typically operationalized as the frequency and total number of psychotherapy sessions completed, represents another factor that may influence symptom change trajectories [10,11]. Classic dose-response models suggest that greater session exposure is generally associated with improved outcomes, while “good-enough” models propose that patients often discontinue treatment once sufficient improvement is achieved, resulting in nonlinear dose-effect relationships [10,11]. In PTSD treatment, intensive trauma-focused programs that deliver EMDR daily or multiple times per day have demonstrated rapid symptom reduction and relatively low dropout rates [12,13]. In routine outpatient practice, however, EMDR is more commonly delivered on a weekly or biweekly basis, and dropout from trauma-focused therapies remains a persistent concern [4]. These findings highlight the need to clarify how treatment intensity operates within usual-care EMDR settings and whether higher session doses confer incremental benefit without compromising engagement.

Despite strong evidence supporting EMDR’s efficacy, including recent individual participant data meta-analyses demonstrating outcomes comparable to or better than other bona fide treatments [14], relatively little is known about how therapeutic alliance and treatment intensity jointly relate to symptom change in real-world EMDR-centered psychotherapy. Most existing evidence derives from randomized controlled trials or highly structured programs, which may not fully capture the variability inherent in routine outpatient practice [3,4,14]. Retrospective cohort studies leveraging routinely collected outcome and process measures provide an opportunity to bridge this evidence-practice gap by examining symptom trajectories and their predictors under naturalistic conditions [5,6].

The present study addressed this gap by examining the relationships among therapeutic alliance, treatment intensity, and PTSD symptom change in a retrospective single-center cohort of adults who completed EMDR-centered psychotherapy in a private outpatient clinic in the Philippines. Using repeated outcome monitoring of PTSD symptom severity at baseline, mid-therapy, and post-treatment, alongside therapeutic alliance assessment, this study evaluated whether therapeutic alliance predicted symptom change, whether treatment intensity independently contributed to improvement, and whether treatment intensity moderated the alliance-outcome relationship. By focusing on routinely collected clinical data from a real-world setting, this study aimed to generate ecologically valid, practice-based evidence to inform adaptive care pathways, treatment and care planning, and alliance-focused clinical strategies in EMDR-centered psychotherapy.

## Materials and methods

Study design and setting

This study used a single-center retrospective cohort design based on routinely collected clinical records from an EMDR-centered outpatient psychotherapy service in a private psychological clinic in Cavite, Philippines. The design enabled examination of associations among therapeutic alliance, treatment intensity, and PTSD symptom change using existing data without altering clinical care or introducing additional participant burden.

Participants and sampling

A consecutive census sampling approach was used, including all eligible cases treated during the study period (2022-2025) rather than a sampled subset. Records were eligible if clients (1) had a clinician-confirmed PTSD diagnosis recorded in the clinical chart using the Diagnostic and Statistical Manual of Mental Disorders, Fifth Edition (DSM-5) criteria, (2) received EMDR-centered psychotherapy (EMDR alone or EMDR with adjunctive interventions), and (3) had sufficient routine outcome data for longitudinal analysis. Cases were excluded if clients (1) discontinued therapy before completing the EMDR-centered treatment episode, (2) had insufficient outcome data (e.g., only one PTSD Checklist for DSM-5 (PCL-5) time point or missing required alliance data), or (3) received non-EMDR psychotherapy exclusively.

Measures

PTSD Checklist for DSM-5 (PCL-5)

PTSD symptoms were assessed using the PCL-5, a 20-item self-report measure scored from 0 (“not at all”) to 4 (“extremely”), with total scores ranging from 0 to 80. The PCL-5 has demonstrated strong psychometric properties and sensitivity to clinical change [15]. In this clinic, the PCL-5 was administered as part of routine outcome monitoring at baseline, mid-therapy, and post-treatment.

Agnew Relationship Measure-5 Item Version (ARM-5)

Therapeutic alliance was measured using the ARM-5, a brief instrument assessing agreement on goals, agreement on tasks, and bond [16]. The ARM-5 was administered at mid-therapy, defined as the assessment obtained nearest the temporal midpoint (session-median) of the treatment episode, as part of routine assessment procedures.

Operational definitions and time windows

To standardize assessments in routine-care records, operational windows were defined as follows: baseline was the closest assessment obtained before or at session 1; mid-therapy was the assessment nearest the temporal midpoint (session-median) of the episode of care; and post-treatment was the final available measure obtained within two weeks of the last session. When multiple measures were available within a window, the assessment closest in time to the window anchor was selected.

Treatment intensity was operationalized as the total number of completed EMDR-centered psychotherapy sessions during the episode of care. Therapy modality was coded as EMDR-only versus EMDR with adjunctive interventions (e.g., cognitive behavioral therapy (CBT)/dialectical behavior therapy (DBT)/acceptance and commitment therapy (ACT)). Because adjunctive therapy was applied based on clinical need, EMDR-only and EMDR+adjunctive cases were treated as contextual treatment descriptors rather than directly comparable treatment groups.

Data collection procedure

A retrospective chart review was performed after ethics approval. Data were extracted into a de-identified database using a structured abstraction form. Extracted variables included demographic and clinical descriptors (age, sex, trauma type, therapy type), treatment characteristics (total completed sessions), PCL-5 scores at baseline/mid/post, and ARM-5 scores at mid-therapy. All data were de-identified prior to analysis, and the linkage key (when applicable) was stored separately with restricted access.

Data analyses

Analyses were conducted using Jamovi v2.6.44.0. Statistical significance was set at α = 0.05. Data were screened for accuracy, outliers, and missingness, and distributional assumptions were evaluated to guide model interpretation. Three analytic aims were tested. (1) Alliance and symptom change. The association between therapeutic alliance and PTSD symptom change was examined using linear regression models with PCL-5 change scores as dependent variables (baseline→mid, baseline→post, mid→post) and ARM-5 scores as predictors. (2) Treatment intensity and symptom change. Dose-response effects were evaluated using linear regression models with PCL-5 change scores as dependent variables and total completed sessions as the predictor. (3) Moderation (alliance × intensity). Moderation was tested using multiple regression with an interaction term (ARM-5 × treatment intensity) predicting PCL-5 change scores. Age, sex, trauma type, and therapy type were included as covariates in adjusted models to account for potential confounding.

Ethical considerations

This study involved secondary analysis of routinely collected clinical data and was considered minimal risk. Ethical clearance was obtained from the Institutional Ethics Review Committee, with a waiver of informed consent granted due to minimal risk, impracticability of re-contacting former clients, and robust confidentiality protections. Data handling complied with relevant ethical standards for confidentiality and privacy (including secure storage, restricted access, and de-identification procedures). The researcher held an administrative role in the clinic; however, clinical governance and record oversight remained with the supervising psychologist, and role separation procedures were implemented to minimize conflicts of interest.

## Results

Descriptive statistics

Therapeutic alliance, measured using the ARM-5, had a mean score of 24.9 with a standard deviation of 2.72. PTSD symptom severity scores, measured using the PCL-5, showed a mean of 43.7 (SD = 2.96) at baseline, 37.8 (SD = 3.23) at mid-therapy, and 30.0 (SD = 4.46) at post-therapy.

Skewness values ranged from −0.582 (treatment intensity) to 0.310 (PCL-5 mid-therapy), with corresponding standard errors of 0.206 across variables. Kurtosis values ranged from −1.22 (age) to 0.159 (PCL-5 post-therapy), each with a standard error of 0.410. Shapiro-Wilk tests of normality yielded W values between 0.916 (treatment intensity) and 0.993 (ARM-5), with associated p-values spanning < 0.001 to 0.699. More so, the variance inflation factor (VIF) and tolerance values were examined to assess multicollinearity. VIF values ranged from 1.00 to 1.01, and tolerance values ranged from 0.986 to 0.998, all of which fall within acceptable limits (VIF < 5, tolerance > 0.20). These results indicate that problematic multicollinearity was unlikely, suggesting that the regression models were not adversely affected by high intercorrelations among predictors.

Therapeutic alliance as a predictor of PTSD symptom changes

The present analysis examined whether therapeutic alliance predicted early improvements in PTSD symptoms during the first half of EMDR-centered treatment. Simple linear regression showed that therapeutic alliance did not predict symptom change from baseline to mid-treatment, with the effect near zero and virtually no variance explained. These indicate that during the early phase of therapy, alliance strength was not meaningfully associated with initial symptom reduction (Table 1).

**Table 1:** Simple linear regression predicting PTSD symptom change (baseline to mid-therapy) from therapeutic alliance. N = 138. R = 0.013, R² = <0.001. PTSD: post-traumatic stress disorder; SE: standard error; B: unstandardized regression coefficient.

Predictor	B	SE	t	p
Intercept	6.12	1.13	5.39	<0.001
Therapeutic alliance	-0.01	0.05	-0.15	0.878

To further examine the role of therapeutic alliance in overall treatment response, a simple linear regression was also conducted to assess whether therapeutic alliance predicted symptom improvement from baseline to post-therapy. Results presented in Table 2 showed that therapeutic alliance was a strong and statistically significant predictor of overall PTSD symptom reduction, accounting for approximately 24.5% of the variance in treatment outcomes. This finding indicates that a stronger therapeutic alliance at mid-therapy was associated with greater reductions in PTSD symptoms by the end of therapy.

**Table 2:** Simple linear regression predicting PTSD symptom change (baseline to post-therapy) from therapeutic alliance. N = 138. R = 0.495, R² = 0.245. PTSD: post-traumatic stress disorder; SE: standard error; B: unstandardized regression coefficient.

Predictor	B	SE	t	p
Intercept	-1.55	2.31	-0.67	0.504
Therapeutic alliance	0.61	0.09	6.65	<0.001

A simple linear regression was also conducted to determine whether therapeutic alliance predicted symptom improvement from mid-therapy to post-therapy. Findings in Table 3 indicated that alliance was a strong and significant predictor of late-phase symptom reduction, accounting for approximately 20.7% of the variance in improvement. These findings suggest that clients who reported higher levels of therapeutic alliance midway through therapy continued to demonstrate greater gains during the latter sessions of EMDR-based therapy.

**Table 3:** Simple linear regression predicting PTSD symptom change (mid-therapy to post-therapy) from therapeutic alliance. N = 138. R = 0.454, R² = 0.207. PTSD: post-traumatic stress disorder; SE: standard error; B: unstandardized regression coefficient.

Predictor	B	SE	t	p
Intercept	-7.67	2.61	-2.93	0.004
Therapeutic alliance	0.62	0.10	5.95	<0.001

Treatment intensity as a predictor of PTSD symptom changes

To examine whether treatment intensity, or the number of completed therapy sessions, predicted early improvements in PTSD symptoms, a multiple regression analysis was conducted, controlling for demographic and clinical characteristics. Table 4 shows that treatment intensity significantly predicted baseline-to-mid-therapy symptom reduction, although the overall effect size was modest. This suggests that clients who attended a greater number of EMDR-centered sessions during the first half of treatment tended to show slightly greater early reductions in PTSD symptoms. None of the control variables, including age, gender, trauma type, or therapy type, were significant predictors of early change (all p > 0.06), indicating that early symptom improvement was driven primarily by treatment intensity rather than demographic or clinical characteristics.

**Table 4:** Multiple regression of treatment intensity predicting PTSD symptom change (baseline to mid-therapy), controlling for demographic and clinical variables. Model fit: R = 0.250, R^2^ = 0.062, F(5, 132) = 1.75, p = 0.127. Note. N = 138. Reference categories are as follows: male (gender), single-incident (trauma type), EMDR-only (therapy type). Outcome variable = ΔPCL-5 (baseline–mid). PTSD: post-traumatic stress disorder; SE: standard error; B: unstandardized regression coefficient; EMDR: eye movement desensitization and reprocessing; PCL-5: PTSD Checklist for DSM-5.

Predictor	B	SE	t	p
Treatment intensity	0.050	0.023	2.13	0.035
Age	-0.003	0.009	-0.38	0.706
Gender	-0.242	0.292	-0.83	0.410
Trauma type	0.463	0.248	1.87	0.064
Therapy type	-0.056	0.269	-0.21	0.836

A multiple regression analysis was also conducted to determine whether treatment intensity predicted overall PTSD symptom change from baseline to post-therapy while controlling for demographic and clinical variables. Results in Table 5 show that treatment intensity was a strong and statistically significant predictor of overall symptom reduction, indicating that clients who completed more EMDR-centered sessions demonstrated greater improvement across the full course of treatment. The final model explained 25.1% of the variance in symptom change and was statistically significant overall. Among the covariates, therapy type emerged as a significant predictor, with clients receiving EMDR only demonstrating significantly greater symptom improvement compared to those receiving EMDR with adjunctive techniques, whereas age, gender, and trauma type were not significant contributors (all p > 0.12).

**Table 5:** Multiple regression of treatment intensity predicting PTSD symptom change (baseline to post-therapy), controlling for demographic and clinical variables. Model fit: R = 0.501, R^2^ = 0.251, F(5, 132) = 8.86, p < 0.001. Note. N = 138. Reference categories: male (gender), single-incident trauma (trauma type), EMDR-only (therapy type). Outcome variable = ΔPCL-5 (baseline–post). PTSD: post-traumatic stress disorder; SE: standard error; B: unstandardized regression coefficient; EMDR: eye movement desensitization and reprocessing; PCL-5: PTSD Checklist for DSM-5.

Predictor	B	SE	t	p
Treatment intensity	−0.213	0.049	−4.36	<0.001
Age	−0.028	0.018	−1.55	0.125
Gender	−0.196	0.613	−0.32	0.750
Trauma type	0.133	0.520	0.26	0.799
Therapy type	−2.409	0.565	−4.27	<0.001

More so, a multiple regression analysis was also conducted to examine whether the number of completed treatment sessions predicted PTSD symptom improvement from mid-therapy to post-therapy, controlling for demographic and clinical factors. Results in Table 6 show that treatment intensity remained a strong and significant predictor of late-phase symptom change, accounting for a meaningful proportion of variance in improvement. This indicates that clients who completed more sessions during the later phase of therapy experienced significantly greater symptom reduction from the mid-point to the conclusion of EMDR-centered therapy. Among the covariates, therapy type was again a significant predictor, with clients who received EMDR-only showing greater improvement than those who received EMDR supplemented with adjunctive techniques. Age, gender, and trauma type did not significantly predict change during this interval (all p > 0.21). Nonetheless, it should be noted that although the EMDR-only group demonstrated greater symptom reduction compared with the EMDR + adjunctive therapy group, this pattern should not be interpreted as evidence that EMDR alone is inherently superior. The present study was not designed as a comparative efficacy trial, and patients who received adjunctive interventions generally presented with greater complexity, higher affective dysregulation, or comorbid concerns that warranted additional skills-based preparation. These clinical characteristics often require more stabilization, a longer therapeutic runway, and more gradual pacing, which can naturally yield slower symptom improvement despite appropriate care. Thus, the observed differences likely reflect initial case severity and treatment needs rather than the relative effectiveness of EMDR versus combined modalities.

**Table 6:** Multiple regression of treatment intensity predicting PTSD symptom change (mid-therapy to post-therapy), controlling for demographic and clinical variables. Model fit: R = 0.498, R^2^ = 0.248, F(5, 132) = 8.73, p < 0.001. Note. N = 138. Reference categories: male (gender), single-incident trauma (trauma type), EMDR-only (therapy type). Outcome variable = ΔPCL-5 (mid-therapy to post-therapy). PTSD: post-traumatic stress disorder; SE: standard error; B: unstandardized regression coefficient; EMDR: eye movement desensitization and reprocessing; PCL-5: PTSD Checklist for DSM-5.

Predictor	B	SE	T	p
Treatment intensity	−0.263	0.054	−4.87	<0.001
Age	−0.025	0.020	−1.24	0.219
Gender	0.046	0.676	0.07	0.946
Trauma type	−0.330	0.574	−0.58	0.566
Therapy type	−2.353	0.623	−3.78	<0.001

Treatment intensity as a moderator of therapeutic alliance and PTSD symptom changes

A moderation analysis was conducted to examine whether treatment intensity altered the association between therapeutic alliance and early PTSD symptom change from baseline to mid-therapy. As shown in Table 7, after controlling for age, gender, trauma type, and therapy type, neither therapeutic alliance nor treatment intensity uniquely predicted early symptom improvement. Most importantly, the interaction between treatment intensity and therapeutic alliance was nonsignificant, indicating that the strength of the alliance was not differentially related to early symptom change at varying levels of treatment intensity. The overall model explained a small proportion of variance and was not statistically significant.

**Table 7:** Regression analysis including the treatment intensity × therapeutic alliance interaction (baseline to mid-therapy). Model fit: R = 0.262, R^2^ = 0.069, F(7, 130) = 1.38, p = 0.218. Note. N = 138. Predictors were mean-centered prior to analysis. Reference categories: male (gender), complex trauma (trauma type), EMDR + adjunctive (therapy type). Outcome variable = ΔPCL-5 (baseline–mid-therapy). SE: standard error; B: unstandardized regression coefficient; EMDR: eye movement desensitization and reprocessing; PCL-5: Post-traumatic Stress Disorder Checklist for DSM-5.

Predictor	B	SE	t	p
Age	−0.004	0.009	−0.49	0.625
Gender	0.240	0.295	0.82	0.417
Trauma type	−0.498	0.251	−1.98	0.050
Therapy type	0.043	0.276	0.16	0.876
Treatment intensity	0.047	0.024	1.97	0.051
Therapeutic alliance	0.003	0.047	0.06	0.950
Treatment intensity × therapeutic alliance	0.008	0.008	0.98	0.327

Another moderation analysis was conducted to determine whether treatment intensity moderated the relationship between therapeutic alliance and overall PTSD symptom improvement from baseline to post-therapy. Table 8 shows that both therapeutic alliance and treatment intensity were strong and significant predictors of overall symptom reduction, indicating that higher alliance and greater treatment dosage independently contributed to better outcomes. Therapy type also emerged as a significant predictor, with clients receiving EMDR only demonstrating greater improvement than those who received adjunctive techniques. In contrast, age, gender, and trauma type were non-significant. Most importantly, the interaction between treatment intensity and therapeutic alliance was not significant, indicating that treatment intensity did not modify the strength of the alliance-outcome relationship. The regression model accounted for a substantial proportion of variance, but this was driven by the strong main effects of alliance and treatment intensity, not their interaction.

**Table 8:** Regression analysis including the treatment intensity × therapeutic alliance interaction (baseline to post-therapy). Model fit: R = 0.631, R^2^ = 0.399, F(7, 130) = 12.30, p < 0.001. Note. N = 138. Predictors were mean-centered prior to analysis. Reference categories: male (gender), complex trauma (trauma type), EMDR + adjunctive (therapy type). Outcome variable = ΔPCL-5 (baseline–post). SE: standard error; B: unstandardized regression coefficient; EMDR: eye movement desensitization and reprocessing; PCL-5: Post-traumatic Stress Disorder Checklist for DSM-5.

Predictor	B	SE	t	p
Age	−0.016	0.017	−0.96	0.341
Gender	−0.090	0.556	−0.16	0.872
Trauma type	−0.006	0.474	−0.01	0.989
Therapy type	1.871	0.521	3.59	<0.001
Treatment intensity	−0.172	0.045	−3.85	<0.001
Therapeutic alliance	0.472	0.089	5.32	<0.001
Treatment intensity × therapeutic alliance	−0.019	0.015	−1.27	0.206

Moreover, a moderation analysis was also conducted to determine whether treatment intensity influenced the relationship between therapeutic alliance and late-phase PTSD symptom improvement from mid-treatment to post-treatment. Results in Table 9 demonstrated that both therapeutic alliance and treatment intensity were significant predictors of symptom reduction, indicating that clients with stronger alliance and those who attended more sessions experienced greater improvement during the later phase of treatment. Therapy type also remained a significant predictor, with EMDR-only clients showing greater gains than those who received adjunctive interventions. In contrast, age, gender, and trauma type did not significantly predict symptom change during this period. Most importantly, the interaction between treatment intensity and therapeutic alliance was not significant, suggesting that treatment intensity did not alter the strength or direction of the alliance-outcome relationship during the mid-to-post-therapy interval. The full model accounted for a substantial proportion of outcome variance, but this was driven exclusively by the strong main effects of alliance, treatment intensity, and therapy type, not their interaction.

**Table 9:** Regression analysis including the treatment intensity × therapeutic alliance interaction (mid-therapy to post-therapy). Model fit: R = 0.615, R^2^ = 0.378, F(7, 130) = 11.30, p < 0.001. Note. N = 138. Predictors were mean-centered prior to analysis. Reference categories: male (gender), complex trauma (trauma type), EMDR + adjunctive (therapy type). Outcome variable = ΔPCL-5 (mid-therapy to post-therapy). SE: standard error; B: unstandardized regression coefficient; EMDR: eye movement desensitization and reprocessing; PCL-5: Post-traumatic Stress Disorder Checklist for DSM-5.

Predictor	B	SE	t	p
Age	−0.012	0.019	−0.62	0.537
Gender	−0.330	0.623	−0.53	0.597
Trauma type	0.492	0.531	0.93	0.357
Therapy type	1.828	0.584	3.13	0.002
Treatment intensity	−0.219	0.050	−4.36	<0.001
Therapeutic alliance	0.469	0.099	4.72	<0.001
Treatment intensity × therapeutic alliance	−0.027	0.017	−1.60	0.112

## Discussion

This retrospective cohort study examined how therapeutic alliance and treatment intensity relate to PTSD symptom improvement in a naturalistic EMDR-centered outpatient setting in the Philippines. Overall, the pattern of findings indicates that PTSD symptoms decreased over the course of treatment and that both therapeutic alliance and treatment intensity were significantly associated with symptom improvement. Importantly, these factors showed complementary patterns of association with symptom change: alliance was most informative once therapy had progressed beyond initial engagement and stabilization, while treatment intensity reflected a dose-related pathway through which clients had a greater opportunity to complete the active components of trauma-focused work. The results support an applied model of EMDR-centered care in which relationship quality and sufficient session “dose” function as parallel processes associated with recovery rather than interchangeable explanations.

A central finding was the temporal nature of therapeutic alliance in predicting outcomes. Alliance did not meaningfully relate to early symptom change, but it was consistently associated with later improvement across the remainder of treatment. This aligns with broader psychotherapy literature showing that alliance is a robust predictor of outcome, particularly when measured after clients and therapists have moved from initial engagement into sustained collaborative work [7,17]. In trauma-focused psychotherapy specifically, early sessions often involve assessment, psychoeducation, stabilization, and safety-building [10,18]; symptom fluctuations during this stage may be influenced by external stressors, ongoing threat, and the client’s initial adjustment to emotionally demanding work, making early alliance ratings less stable and less predictive [10]. Consistent with Zilcha-Mano’s (2017) conceptualization of alliance as containing both trait-like and state-like components, alliance assessed later may better capture a consolidated working bond, one that reflects genuine collaboration on goals and tasks and a secure therapeutic base that supports deeper trauma processing [18].

Within an EMDR framework, the finding that alliance becomes more prognostically meaningful later in treatment is also clinically coherent. EMDR protocols commonly require adequate stabilization and preparation before clients fully engage in memory reprocessing, and it is often during or after sustained reprocessing phases that larger changes in symptom severity emerge [19]. As clients progress toward the more emotionally intense elements of EMDR, alliance may serve as a key relational “container” that supports dual attention, tolerable affect activation, and persistence through distressing material [7,9,18,19]. In this view, alliance does not necessarily produce immediate symptom relief early on; rather, it may enable the depth and continuity of therapeutic work required for later gains, an interpretation consistent with alliance-outcome models in trauma-focused treatment [7,9,18].

Treatment intensity also emerged as a meaningful predictor of symptom improvement, with the strongest contribution observed later in treatment. This pattern supports long-standing dose-response findings in psychotherapy, wherein greater exposure to treatment is generally associated with greater clinical change [9,11]. In EMDR-centered care, attending more sessions likely increases opportunities for multiple reprocessing targets, installation of adaptive cognitions, and consolidation of gains, all of which are theorized to be central mechanisms of symptom reduction [19]. The comparatively smaller association between intensity and early change is also consistent with typical clinical sequencing: early sessions may be devoted to case conceptualization, resourcing, and skills-building, whereas later sessions increasingly feature active trauma processing and integration, which may yield more visible symptom improvement [10,11,19].

Therapy type also showed a consistent pattern, with EMDR-only cases exhibiting greater symptom improvement than cases receiving EMDR plus adjunctive interventions. This finding should be interpreted cautiously as a naturalistic indicator rather than evidence of comparative superiority. In routine care, adjunctive approaches such as CBT, DBT-informed skills work, or exposure-based elements are commonly introduced when clients present with higher complexity, affective dysregulation, comorbidity, or safety concerns, factors that can necessitate longer stabilization and slower pacing [19,20]. As a result, the observed difference likely reflects baseline case complexity and clinical indication rather than a true “treatment purity” advantage. Nonetheless, the pattern is compatible with evidence that structured, protocol-consistent trauma-focused interventions can be highly effective when clients are clinically ready for direct trauma processing, and that deviations or additions may reflect warranted clinical tailoring rather than optimization [19,20].

Notably, treatment intensity did not moderate the relationship between therapeutic alliance and symptom change across therapy phases. Instead, alliance and intensity functioned as additive predictors: both mattered, but the benefit associated with a strong alliance did not depend on session dose, and the benefit associated with more sessions did not depend on alliance level. This is theoretically consistent with contemporary views that alliance is a general, trans-theoretical process factor that supports engagement, emotional safety, and collaborative work, whereas dose represents an exposure-based parameter that increases opportunities for therapeutic mechanisms to unfold [7,18]. Clinically, this pattern implies that maximizing outcomes does not require choosing between relationship-building and ensuring treatment completion; rather, both should be cultivated in parallel. A strong alliance may help retain clients and enable challenging trauma work, while adequate session completion may allow clients to progress through reprocessing and consolidation phases that produce durable symptom improvement [9,19].

Demographic and trauma-related variables were not consistent predictors of symptom change once process and treatment factors were considered. This suggests that, within this cohort, improvement was more strongly tied to modifiable therapy processes (alliance quality, attendance, and clinical decision-making around treatment structure) than to client background characteristics. This does not imply that demographics and trauma complexity are clinically irrelevant; rather, it highlights that EMDR-centered outcomes in routine care may be particularly sensitive to how therapy is delivered and sustained, and to whether a collaborative working bond is established as treatment progresses [7,17]. In practical terms, clinics may gain more by strengthening measurement-based monitoring of alliance and engagement, and by reducing barriers to consistent attendance, than by assuming that certain demographic groups are inherently less likely to benefit.

Several implications follow for trauma-focused practice in real-world Philippine clinical settings. First, alliance should be actively supported beyond the early sessions, especially as clients transition into deeper trauma processing [7,18]. Monitoring alliance mid-treatment may be more clinically informative than relying on early impressions, consistent with alliance theory emphasizing stability and rupture-repair processes over time [18]. Second, the importance of treatment intensity reinforces the need for retention-oriented care: psychoeducation about the expected trajectory of change, collaborative planning around practical barriers, and flexible scheduling may help clients complete enough sessions to move through the core phases of EMDR-centered treatment [9,11,19]. Third, the adjunctive therapy pattern supports careful documentation of clinical rationale and staging, particularly in complex trauma presentations, so that slower symptom change is understood as part of appropriate pacing rather than as treatment failure [20].

Finally, these findings should be interpreted within the constraints of a single-center, retrospective cohort relying on routine measures rather than experimental assignment. Naturalistic designs strengthen ecological validity but also limit causal inference; unmeasured factors such as baseline severity, dissociation, therapist effects, fidelity, and life stressors may have influenced both treatment planning (e.g., adjunctive use) and outcomes. Even so, the overall convergence of the present pattern with established alliance and dose-response theories supports the clinical plausibility of the conclusions: therapeutic alliance appears most consequential once consolidated, treatment intensity reflects a meaningful “dose” pathway for EMDR-centered work, and these two drivers operate alongside each other rather than synergistically [7,9-11,17-20].

## Conclusions

In this retrospective cohort study of adults receiving EMDR-centered psychotherapy in routine outpatient care, both therapeutic alliance and treatment intensity emerged as independent predictors of PTSD symptom improvement. Symptom reduction increased progressively across treatment, with therapeutic alliance demonstrating its strongest association with outcomes after the initial phase of therapy, while greater session completion supported sustained improvement across later stages. These findings suggest that symptom change in real-world trauma-focused psychotherapy reflects the combined influence of relational and dose-related processes rather than early engagement alone.

From a clinical perspective, the results underscore the importance of monitoring and strengthening the therapeutic alliance beyond early sessions, particularly as treatment progresses into more demanding phases of trauma processing. At the same time, promoting treatment retention and sufficient session exposure appears critical for achieving meaningful and durable symptom reduction. These findings support a practice-based model of care that prioritizes both relational quality and continuity of treatment to optimize outcomes in outpatient trauma-focused psychotherapy.
